# A triad of hypertension, heart failure, and glomerular injury in subacute Kawasaki disease: a case report and literature review

**DOI:** 10.3389/fcvm.2026.1804390

**Published:** 2026-05-13

**Authors:** Qian Liu, Jing Liu, Ting Kang, Yuan Long

**Affiliations:** Department of Emergency, Wuhan Children’s Hospital, Tongji Medical College, Huazhong University of Science & Technology, Wuhan, Hubei, China

**Keywords:** endothelial dysfunction, glomerular injury, heart failure, hypertension, Kawasaki disease

## Abstract

Kawasaki disease (KD) is the leading cause of acquired pediatric heart disease, characterized by acute systemic vasculitis. We report a 7-year-6-month-old boy with typical KD [meeting the 2017 American Heart Association (AHA) diagnostic criteria] who responded completely to initial intravenous immunoglobulin (IVIG) therapy (2 g/kg), with rapid defervescence and normalized inflammatory markers. Despite this favorable initial response and no coronary abnormalities, he uniquely developed a triad of acute heart failure with cardiomegaly [New York Heart Association (NYHA) class II], severe hypertension (> 99th percentile), and early glomerular injury (elevated urinary albumin-to-creatinine ratio, urinary transferrin, and urinary albumin) during the clinically quiescent subacute phase. This “inflammatory-silent but vascular-loud” phenotype underscores that effective initial treatment and normalization of inflammatory markers do not equate to vascular safety, highlighting persistent endothelial dysfunction as a critical driver of morbidity. The case supports redefining subacute-phase monitoring to include blood pressure surveillance, comprehensive echocardiographic assessment of chamber dimensions and function, and screening for early glomerular injury.

## 1 Introduction

Kawasaki disease (KD) is an acute, systemic, immune-mediated vasculitis of unknown origin and the leading cause of acquired heart disease in children in developed countries (1). While its classic presentation involves fever, mucocutaneous signs, and lymphadenopathy, the core pathology targets the vascular endothelium, resulting in coronary artery lesions and long-term cardiovascular sequelae (1, 2). The classic clinical course is divided into three phases based on clinical and pathological features: the acute phase (typically lasting 1–2 weeks), characterized by fever and acute inflammatory manifestations; the subacute phase (approximately weeks 2–4), marked by resolution of fever, thrombocytosis, and desquamation; and the convalescent phase (usually 6–8 weeks after disease onset), during which inflammatory markers gradually return to normal (1–3). The disease is understood as a dysregulated immune response in genetically susceptible hosts, evolving from innate immunity hyperactivation to dysfunctional adaptive responses (4). This immunopathology is marked by intense vascular infiltration, Th17/Treg imbalance (5), autoantibody production, and a cytokine storm involving interleukin-1β (IL-1β), IL-6, and tumor necrosis factor-α (TNF-α) (3). Crucially, vascular immune activation can persist locally—sustained by endothelial adhesion molecule expression and pro-fibrotic pathways like transforming growth factor-β (TGF-β)—even after systemic inflammatory markers normalize (6).

We report a case of a 7-year-6-month-old male who, after standard intravenous immunoglobulin (IVIG) treatment for acute KD with resolved systemic inflammation and normal coronary arteries, developed a severe triad during the subacute phase: hypertension, acute heart failure with cardiomegaly, and early glomerular injury. This rare presentation occurring in a state of “systemic inflammatory quiescence” provides critical insights into KD's transition from acute vasculitis to chronic endothelial dysfunction and vascular remodeling.

## 2 Case report

### 2.1 First hospitalization (illness day 1–9)

**Figure 1:**
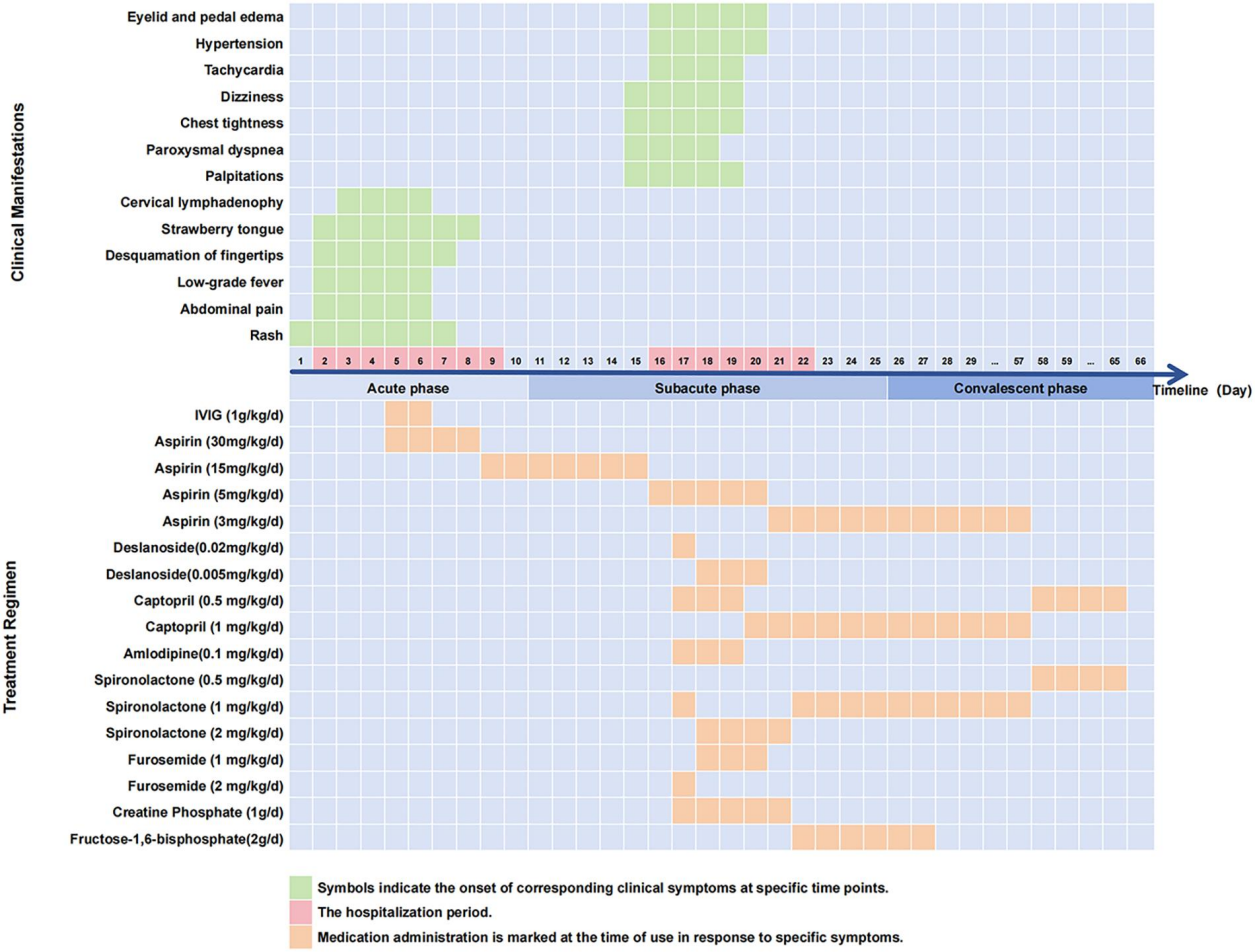
Clinical course and treatment timeline.

A 7-year-6-month-old boy (height 130 cm, weight 29 kg) presented with rash and abdominal pain for 1 day and was admitted on illness day 2. The rash was miliary with partial confluence and mild pruritus; abdominal pain was paroxysmal, periumbilical, without diarrhea. No prodromal fever, cough, or rhinorrhea was reported. Past medical and family histories were unremarkable. Admission vital signs: temperature 37.9 °C, heart rate 95/min, respiratory rate 23/min, blood pressure 93/60 mmHg, oxygen saturation 98%. Physical examination revealed mild pharyngeal congestion, strawberry tongue, coarse breath sounds, regular heart rhythm, soft abdomen with mild periumbilical tenderness, scattered maculopapular rash on the chest and back, and mild fingertip desquamation. From illness day 2 to 6, the patient developed persistent low-grade fever (37.2 °C–38.2 °C), progressive rash, intermittent abdominal pain, mild indurative edema of the fingertips, and a palpable left cervical lymph node (approximately 1.5 cm × 2 cm, soft, non-tender, unilateral). Bilateral conjunctival injection was not observed. Laboratory tests on day 2 showed leukocytosis, elevated high-sensitivity C-reactive protein (hs-CRP 9.24 mg/L), and normal erythrocyte sedimentation rate (ESR 5 mm/h). By day 5, hs-CRP had increased to 124.10 mg/L and ESR to 45 mm/h (detailed results in Table 1). Echocardiography on days 2 and 9 demonstrated normal cardiac chamber dimensions and coronary artery diameters (see Table 2 for detailed parameters). Z-scores were calculated using the Boston Children's Hospital Z-score calculator (https://zscore.chboston.org/), based on the regression equations by Pettersen et al. (7). Electrocardiography (ECG) showed sinus rhythm without repolarization abnormalities (Supplementary Materials). According to the 2017 American Heart Association diagnostic criteria (1), the patient met the criteria for complete KD. IVIG (1 g/kg) was administered on days 5 and 6, respectively, and aspirin 30 mg/kg/day was initiated concurrently (see Figure 1 for detailed dosing). The patient became afebrile by day 7 and was discharged on day 9 on aspirin 15 mg/kg/day, with planned follow-up in one week for dose adjustment. The complete clinical course and treatment timeline are illustrated in Figure 1.

**Table 1:** Laboratory findings.

Index	WBC	PLT	HGB	hs-CRP	ESR	IL-6	TNF-α	ALT	AST	ALB	Urine WBCs	CK-MB	TNT-HS	NT-proBNP
RI	4.6–11.9	177–446	121–158	0∼3	0–15	0–20	0–5.5	7–30	14–44	39–54	Negative	0–25	0–0.014	0–125.2
Unit	10^9/L	10^9/L	g/L	mg/L	mm/h	pg/mL	pg/mL	U/L	U/L	g/L	/HPF	U/L	ng/mL	pg/mL
D2	20.07	289	139	9.24	5	68.59	15.58	13	28	37.9	Negative	23.3	0.004	314.9
D5	12.77	205	117	124.10	45	69.89	16.89	–	–	–	–	–	–	–
D7	5.86	223	109	44.90	40	18.92	10.86	–	–	–	–	–	–	–
D8	6.05	250	110	29.19	20	2.56	5.62	–	–	–	–	–	–	–
D9	7.30	237	125	17.78	15	1.56	2.35	7	21	32.5	Negative	9.40	0.005	299.8
D16	6.77	269	104	1.26	10	0.87	1.01	16	31	39.5	0–1	14.5	0.013	11596.0
D18	7.05	256	110	1.03	10	0.83	0.89	–	–	–	–	18.4	0.008	9800.0
D20	5.08	536	113	2.78	–	–	–	11	25	40.5	Negative	19.5	0.007	3000.0
D22	6.03	251	121	1.03	–	–	–	–	–	–	–	16.1	0.008	497.0
D28	6.55	223	125	1.89	–	–	–	–	–	–	–	–	–	102.0
D58	7.15	241	132	1.67	–	–	–	–	–	–	–	–	–	64.0

WBC, white blood cell (count); PLT, platelet (count); HGB, hemoglobin; hs-CRP, high-sensitivity C-reactive protein; ESR, erythrocyte sedimentation rate; IL-6, interleukin-6; TNF-α, tumor necrosis factor-alpha; ALT, alanine aminotransferase; AST, aspartate aminotransferase; ALB, albumin, urine WBCs, urine white blood cells; CK-MB, creatine kinase-myocardial band; TNT-HS, high-sensitivity troponin T; NT-proBNP, N-terminal pro-B-type natriuretic peptide; RI, reference interval. D1 is defined as the first day following the onset of clinical symptoms, and the numbering continues accordingly for subsequent days (D2, D3…).

**Table 2:** Echocardiographic findings.

Index	LVEDD		LA (AD)		RV	RA	LCA		LAD		RCA		EF	FS
	Measured	Z-score	Measured	Z-score	Measured	Measured	Measured	Z-score	Measured	Z-score	Measured	Z-score		
D2	37 mm	−1.38	19 mm	−1.77	29 mm	30 mm	2.8 mm	−0.27	2.6 mm	+0.76	1.8 mm	−1.5	62%	32%
D9	43 mm	+1.04	24 mm	−0.06	31 mm	31 mm	3.0 mm	+0.16	2.4 mm	+0.13	1.8 mm	−1.5	64%	33%
D16	48 mm	+3.07	33 mm	+2.27	36 mm	32 mm	3.0 mm	+0.16	2.9 mm	+1.7	2.6 mm	+0.53	51%	26%
D19	46 mm	+2.26	28 mm	+1.06	34 mm	32 mm	3.1 mm	+1.38	2.8 mm	+0.37	2.4 mm	+0.02	54%	28%
D22	42 mm	+0.64	25 mm	+0.24	31 mm	32 mm	3.2 mm	+0.59	2.5 mm	+0.44	2.1 mm	−0.74	61%	32%
D28	42 mm	+0.64	24 mm	−0.06	31 mm	33 mm	2.8 mm	−0.27	2.5 mm	+0.44	2.0 mm	−0.99	60%	32%
D58	43 mm	+1.04	23 mm	−0.37	32 mm	32 mm	2.7 mm	−0.49	2.2 mm	−0.5	1.9 mm	−1.25	60%	32%

LVEDD, left ventricular end-diastolic dimension; LA (AD), left atrium (anteroposterior dimension); RV, right ventricle; RA, right atrium; LCA, left coronary artery; LAD, left anterior descending artery; RCA, right coronary artery; EF, ejection fraction; FS, fractional shortening. D1 is defined as the first day following the onset of clinical symptoms, and the numbering continues accordingly for subsequent days (D2, D3…).

### 2.2 Second hospitalization (illness day 16–22)

On illness day 15, the patient suddenly developed palpitations, paroxysmal dyspnea, chest tightness, and dizziness, prompting readmission the following day (day 16). On admission, the patient was afebrile (36.8 °C), tachycardic (140/min), and hypertensive (160/110 mmHg, > 99th percentile), with a respiratory rate of 24/min and SpO_2_ 97%. Physical examination revealed mild pitting edema of the eyelids and dorsa of both feet; the rash had completely resolved, and fingertip desquamation had improved. Laboratory tests showed normal inflammatory markers (hs-CRP, ESR) and cardiac enzymes [creatine kinase-myocardial band (CK-MB), high-sensitivity troponin T (TNT-HS)], but N-terminal pro-B-type natriuretic peptide (NT-proBNP) was markedly elevated to 11596.0 pg/mL (peak value on admission; see Table 1 for detailed trends). Renal function was normal; however, urinalysis revealed elevated albumin-to-creatinine ratio (ACR) of 13.75 mg/mmol (normal < 3), urinary transferrin (U-TRF) of 11.4 mg/L (normal < 2), and urinary albumin (UALB) of 133.1 mg/L (normal < 30). Immunologic and endocrine workups [anti-neutrophil cytoplasmic antibodies (ANCA), anti-glomerular basement membrane (anti-GBM), antinuclear antibody (ANA), renin-angiotensin-aldosterone system (RAAS), thyroid function] were unremarkable. ECG demonstrated atrial tachycardia without repolarization abnormalities (no ST-segment or T-wave changes). Echocardiography on day 16 showed normal coronary arteries, left ventricular and atrial dilation, and mildly reduced systolic function [ejection fraction (EF) 51%]; Z-scores are provided in Table 2. Twenty-four-hour ambulatory blood pressure monitoring (ABPM) confirmed sustained hypertension: peak systolic blood pressure (SBP) 169 mmHg, peak diastolic blood pressure (DBP) 122 mmHg, daytime average 160/98 mmHg (> 99th percentile).

Based on these findings, the patient was diagnosed with KD (subacute phase), acute heart failure [New York Heart Association (NYHA) class II] (8), secondary hypertension [stage 2, per 2017 American Academy of Pediatrics (AAP) guidelines (9)], atrial tachycardia, and early glomerular injury (10). Treatment was initiated with amlodipine and captopril concurrently for better blood pressure control, along with deslanoside, furosemide, and spironolactone. The treatment timeline is illustrated in Figure 1.

Following combination therapy, the patient showed progressive improvement. On day 18, NT-proBNP decreased to 9800.0 pg/mL; urinary ACR was 14.89 mg/mmol, U-TRF 12.4 mg/L, and UALB 120.5 mg/L. Echocardiography on day 19 demonstrated reduced chamber dilation and improved EF (54%; see Table 2). Atrial tachycardia terminated by day 20, and blood pressure normalized by day 21, with complete resolution of edema. On day 22, prior to discharge, echocardiography revealed normalized chamber dimensions and systolic function, with no coronary dilation (Table 2). Cardiac computed tomography angiography confirmed resolution of left heart enlargement and normal coronary arteries. On discharge, NT-proBNP had decreased to near-normal levels, and urinary markers returned to normal. Discharge medications included aspirin, captopril, and spironolactone (dosing timeline in Figure 1). Serial changes in cardiac chamber sizes and ECGs are available in the Supplementary Materials.

### 2.3 Follow-up (illness day 23–66)

The patient was followed regularly after discharge. Echocardiography and NT-proBNP remained normal at both the day 28 and day 58 follow-up visits. Aspirin was discontinued on day 58, and captopril and spironolactone were discontinued on day 65. At the last follow-up on day 66, the patient remained asymptomatic with no recurrence of symptoms. Detailed echocardiographic parameters are provided in Table 2, and serial changes in cardiac chamber sizes are illustrated in the Supplementary Materials.

## 3 Discussion

The essence of this case lies in the concurrent development of cardiomegaly, severe hypertension, and early glomerular injury during the subacute phase of KD, occurring in the context of fully normalized systemic inflammatory markers. This “triad” is exceedingly rare in the published literature. Its underlying mechanism cannot be adequately explained by the conventional acute inflammation paradigm and instead requires re-evaluation within the conceptual framework of “sustained dysregulation of the immune-vascular axis”.

### 3.1 Clinical uniqueness

Hypertension associated with KD is primarily observed during long-term follow-up, with the risk being 2.2 times higher than in controls in adults (11). Hemodynamic abnormalities in the acute phase are typically seen in Kawasaki disease shock syndrome (KDSS), presenting as hypotension due to hypovolemia or myocardial suppression (12). In contrast, our patient developed isolated severe hypertension with heart failure during the subacute phase, a rare presentation. KD-related kidney injury is usually linked to KDSS, rhabdomyolysis, or nephrotoxic drugs, often with a hyperinflammatory state. Here, no shock, elevated muscle enzymes, nephrotoxic exposure, or systemic inflammation was present, yet glomerular barrier injury (microalbuminuria) occurred, indicating endothelium-specific damage. Myocarditis is common in acute KD, with subclinical myocarditis confirmed by endomyocardial biopsy (13); myocardial injury typically parallels inflammation, manifesting as decreased EF and elevated TNT-HS (14). In this case, TNT-HS was negative while NT-proBNP was markedly elevated, consistent with pressure-overload ventricular dilation rather than necrosis (13). Compared with prior reports, this case is distinct: Choi et al. described an IVIG-nonresponsive KDSS child with hypertension and acute kidney injury, but kidney injury occurred during shock, with hypertension appearing later (14); Krug et al. reported three KD cases with nephrotic syndrome where proteinuria resolved within 1–2 weeks without hypertension or cardiomegaly (15); Kim et al. reported refractory incomplete KD with acute myocardial infarction in the subacute phase, but the patient was IVIG-nonresponsive with progressive coronary dilation (16); Zhang et al. reported infant KD with supraventricular tachycardia that paralleled acute inflammation and resolved with its control (17). Our patient uniquely developed the triad—heart failure, hypertension, and early glomerular injury—during the subacute phase despite being IVIG-sensitive, having normalized inflammatory markers, and normal coronary arteries. This phenotype, absent from current guidelines, suggests persistent pathogenic mechanisms beyond traditional inflammation.

### 3.2 Hypothesis: endothelial dysfunction drives immune-vascular axis dysregulation

**Figure 2:**
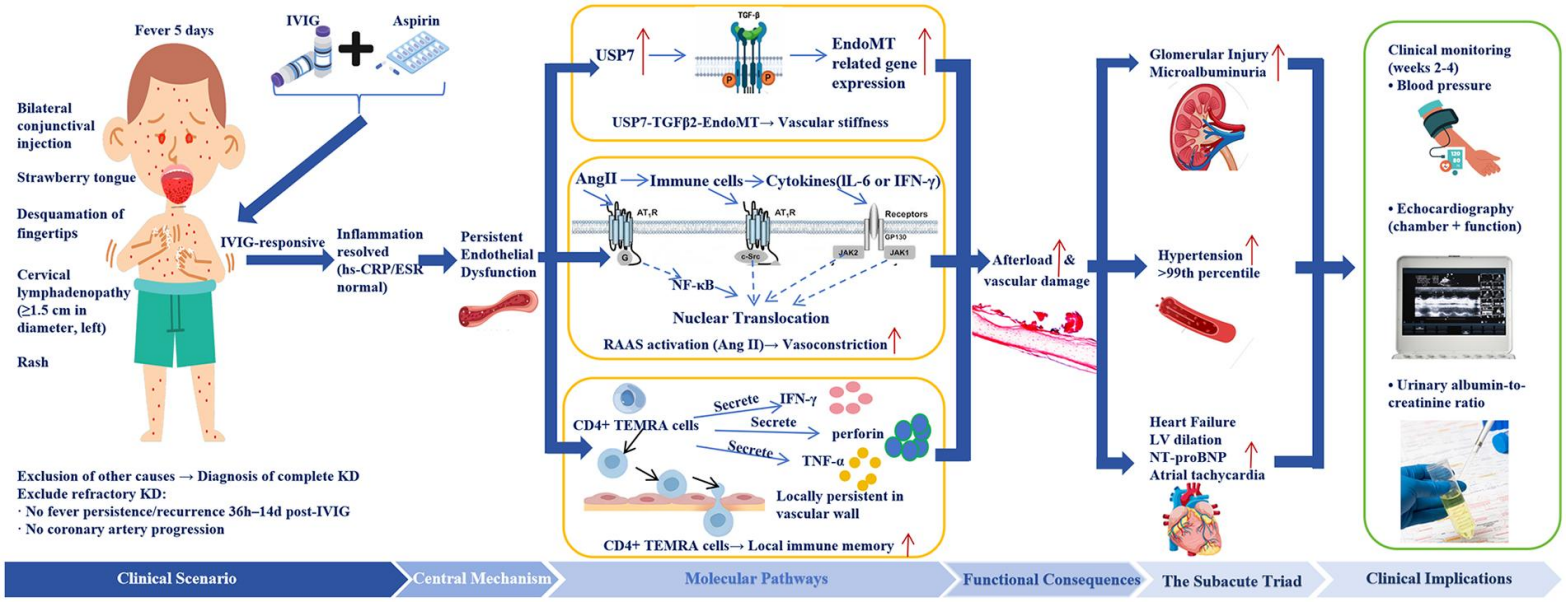
Integrated clinical-mechanistic-management model of the subacute cardiovascular-renal triad in KD. hs-CRP, high-sensitivity C-reactive protein; ESR, erythrocyte sedimentation rate; IVIG, intravenous immunoglobulin; KD, Kawasaki disease; RAAS, renin-angiotensin-aldosterone system; TEMRA, terminally differentiated effector memory T cells; LV, left ventricle; NT-proBNP, N-terminal pro-B-type natriuretic peptide.

We hypothesize that the concurrent development of hypertension, cardiomegaly, and early glomerular injury following systemic inflammation resolution reflects parallel manifestations of persistent systemic endothelial dysfunction. This hypothesis is supported by multidimensional clinical evidence: microalbuminuria, a marker of glomerular endothelial injury, is a sensitive indicator of systemic endothelial dysfunction (18, 19); severe hypertension (> 99th percentile) aligns with endothelium-dependent vasodilatory dysfunction involving reduced nitric oxide bioavailability (20, 21); cardiomegaly with elevated NT-proBNP but normal troponin is consistent with pressure-overload remodeling from increased afterload; atrial tachycardia synchronized with left atrial diameter changes (33 mm to 28 mm) and left atrial origin on ECG supports atrial stretch as a trigger; and the therapeutic response to captopril—which inhibits angiotensin II and enhances bradykinin-mediated nitric oxide release—indirectly supports endothelial dysfunction as central (20, 21). Even after circulating inflammatory markers decline, endothelial cells may sustain pro-inflammatory gene “memory” via epigenetic modifications such as H3K27ac enrichment. Persistent activation of the ubiquitin-specific protease 7 (USP7)-TGF-β2-SMAD pathway can drive endothelial-to-mesenchymal transition (EndoMT), endowing endothelial cells with fibroblastic properties that secrete collagen and promote vascular stiffness without elevating CRP (6, 22). This focal, non-inflammatory vascular remodeling directly reduces compliance and increases afterload. Furthermore, the RAAS exerts immunoregulatory effects: angiotensin II not only vasoconstricts but also activates nuclear factor-κB (NF-κB) via angiotensin type 1 (AT1) receptors, promoting monocyte chemotaxis and T-cell infiltration (23). Locally persistent terminally differentiated effector memory T cells (CD4⁺ TEMRA) can maintain low-grade immune activity in the vascular wall (24). The rapidly progressive hypertension in our patient likely stems from KD-induced RAAS activation, supported by captopril's efficacy. These mechanisms continuously impair vascular function without systemic inflammation, exemplifying “occult vascular immunopathology”. While this hypothesis requires prospective validation, the convergence of clinical and molecular evidence offers a testable framework for understanding subacute cardiovascular complications in KD. Figure 2 schematically summarizes the key pathogenic pathways, including persistent endothelial dysfunction driven by EndoMT, RAAS activation, and CD4⁺ TEMRA-mediated local immunity, and their translation into subacute target organ damage.

### 3.3 Clinical implications: redefining monitoring strategies for the subacute phase

This case carries important clinical implications. According to the 2017 AHA guidelines, refractory KD is defined by fever persistence after IVIG, not by complications (1). Our patient defervesced within 36 h post-IVIG, yet developed the triad in the subacute phase despite normalized inflammatory markers. This underscores that IVIG sensitivity and fever resolution do not guarantee cardiovascular safety; local vascular pathology may progress independently of systemic inflammation. Endothelial dysfunction thus emerges as a core pathway linking acute KD to long-term sequelae. Future strategies should extend beyond acute inflammation control to endothelial protection and inhibition of vascular remodeling. We strongly recommend routine monitoring for all KD children during weeks 2–4 of illness, irrespective of acute severity: rigorous blood pressure monitoring; detailed echocardiography assessing not only coronary arteries but also cardiac chamber sizes and ventricular function; and screening for early renal injury via urinary microalbumin-to-creatinine ratio (18). For those with abnormalities, early introduction of RAAS inhibitors (e.g., captopril) may be considered. Complementing the mechanistic overview above, Figure 2 also incorporates the diagnostic workup (excluding other diseases and refractory KD) and the proposed monitoring strategy.

## 4 Conclusion

This report highlights that KD can progress as a smoldering endothelial disorder beyond the acute inflammatory phase. The presented triad—cardiomegaly, hypertension, and early glomerular injury—exemplifies significant multi-organ dysfunction driven by persistent immune-mediated vascular injury, despite normalized systemic inflammation and intact coronary arteries. This necessitates a paradigm shift in KD management from a focus solely on suppressing acute inflammation to one that actively monitors and addresses sustained endothelial dysfunction and vascular remodeling. Integrating vascular health assessment into long-term follow-up is critical for advancing precision medicine in KD.

## Data Availability

The original contributions presented in the study are included in the article/Supplementary Material, further inquiries can be directed to the corresponding author.
