# Supplementary material for: A triad of hypertension, heart failure, and glomerular injury in subacute Kawasaki disease: a case report and literature review
Source: Front Cardiovasc Med. 2026 May 13;13:1804390. doi: 10.3389/fcvm.2026.1804390 (PMC13212307; doi:10.3389/fcvm.2026.1804390)
Supplement: Supplementary file 1 [file Datasheet1.pdf]

D2 HR 132bpm ↑[70~110]

PR 108ms QRSD 83ms [40~100]
QT 292ms QTc 433ms [0~440]
QRS 76° [-22~115] RV5/SV1 1.825/1.734mv

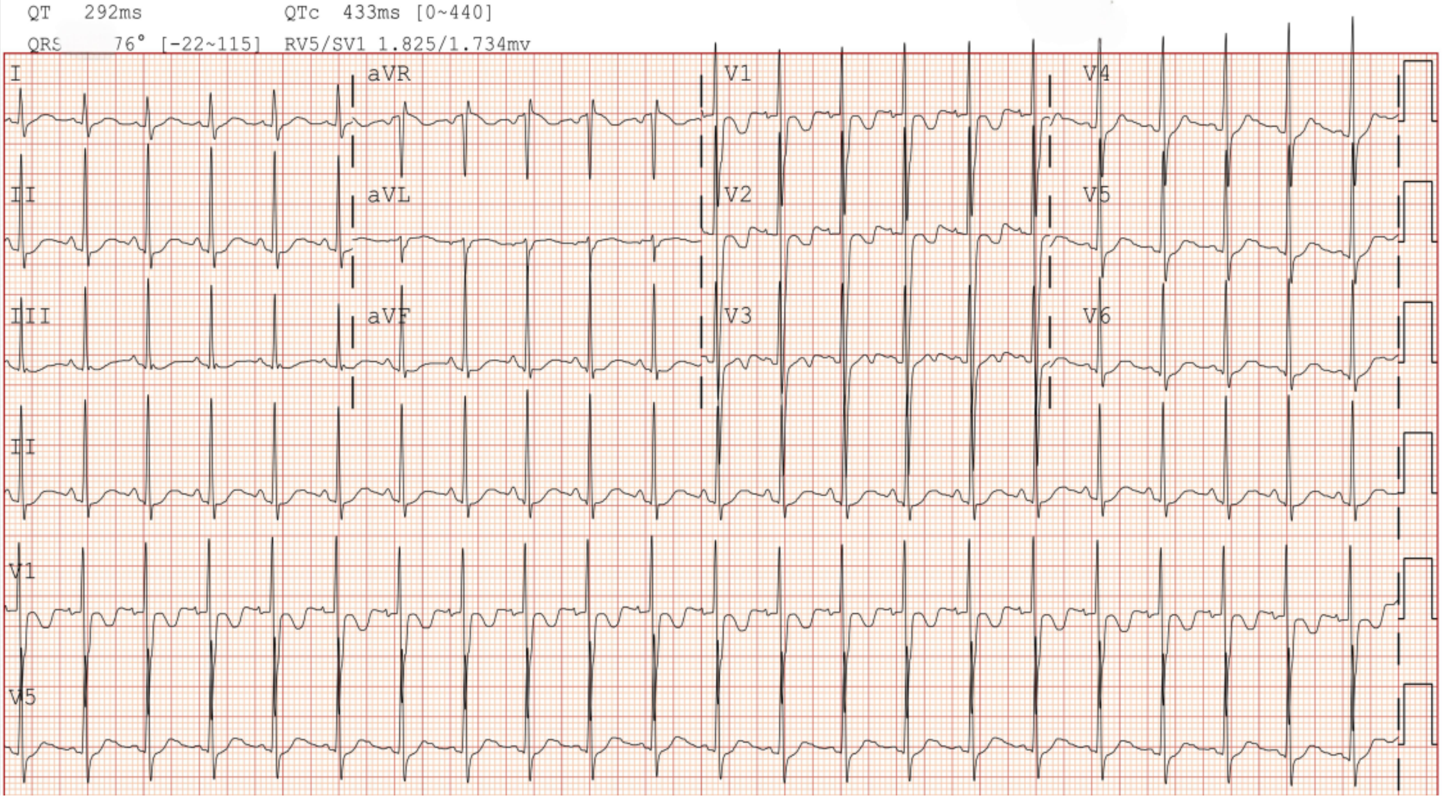

D16 HR 126bpm ↑[70~110]

PR 123ms [110~140] QRSD 84ms [40~100]
QT 275ms QTc 399ms [0~440]
QRS 92° [-22~115] RV5/SV1 3.456/0.858mv

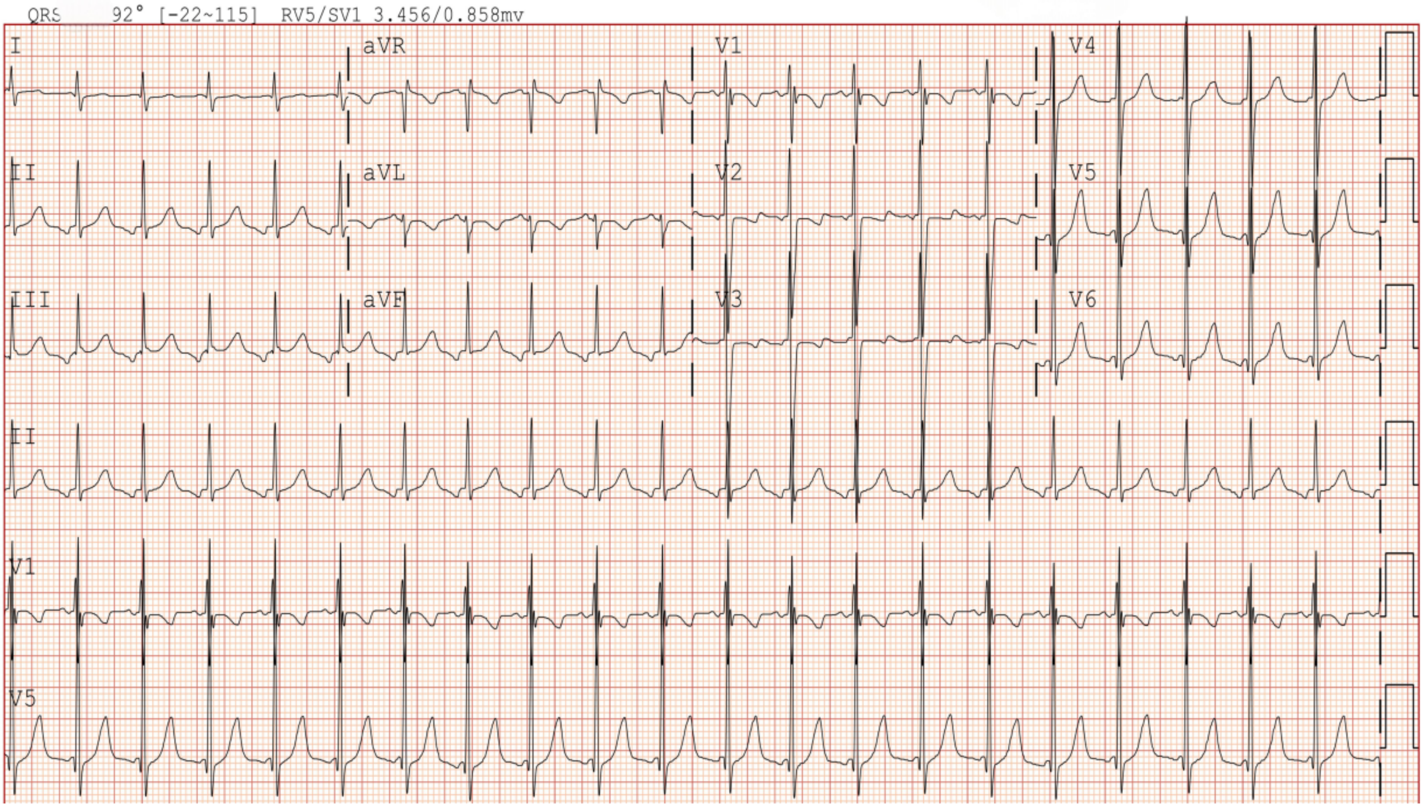

D17 HR 124bpm ↑[70~110]

PR 137ms [110~140] QRSD 82ms [40~100]
QT 296ms QTc 425ms [0~440]
QRS 86° [-22~115] RV5/SV1 3.259/0.891mv

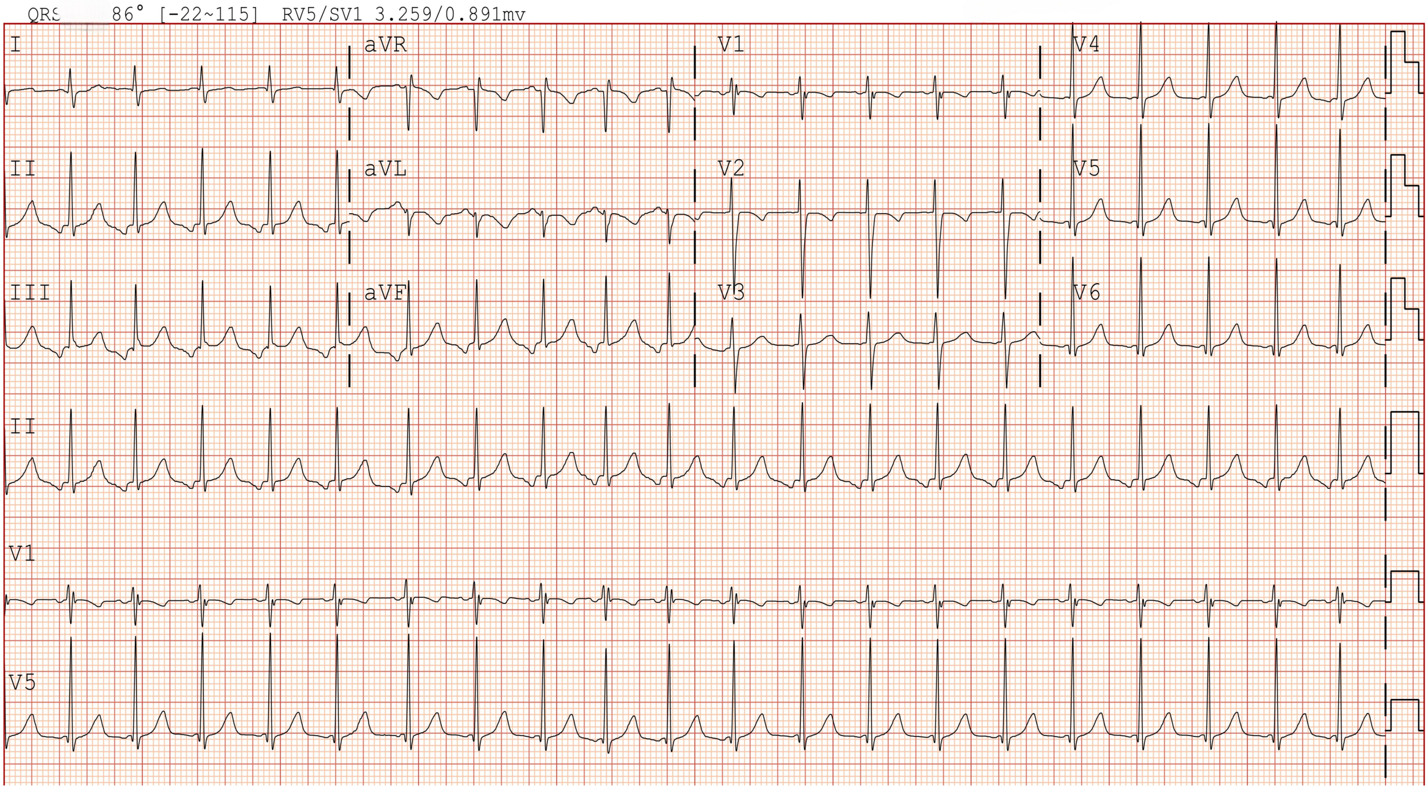

D20 HR 61bpm ↓[70~110]
PR 123ms QRSD 87ms [40~100]
QT 385ms QTc 382ms [0~440]
QRS 55° [-22~115] RV5/SV1 2.538/0.747mv

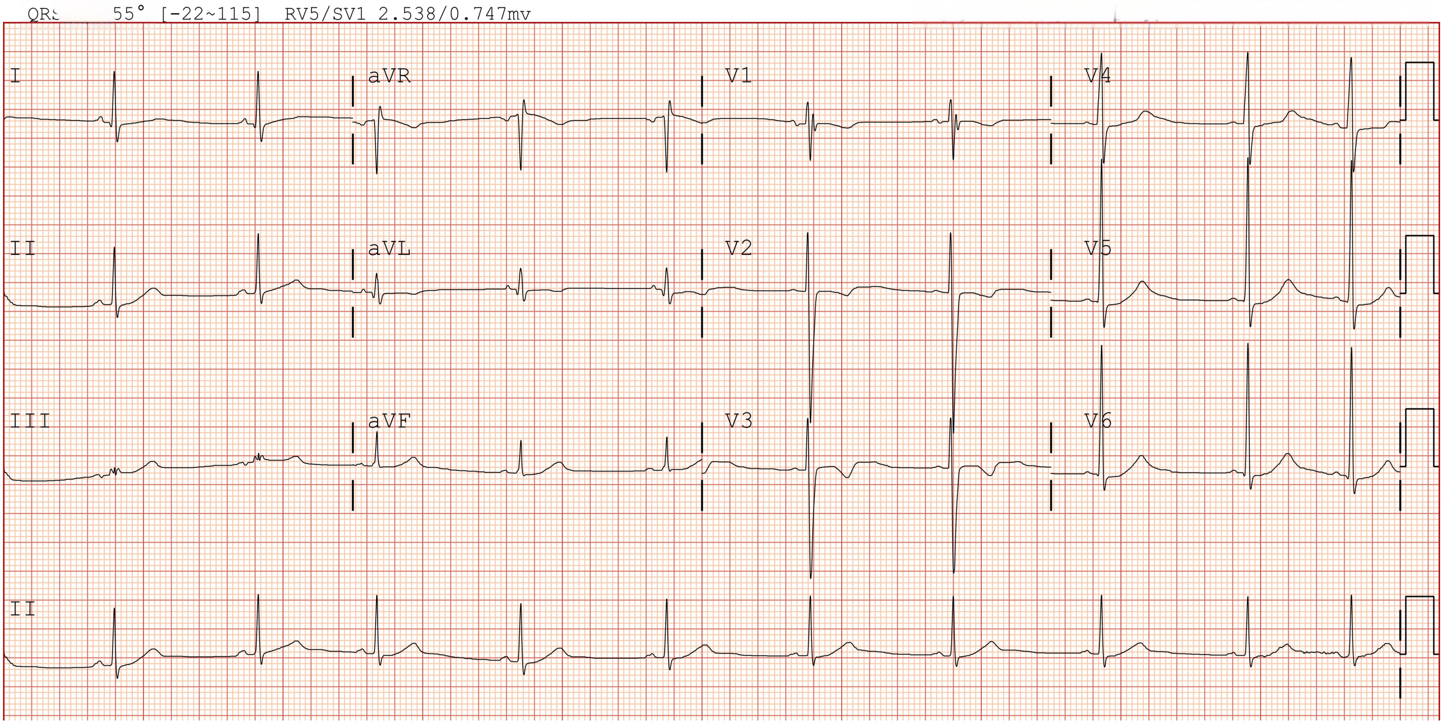

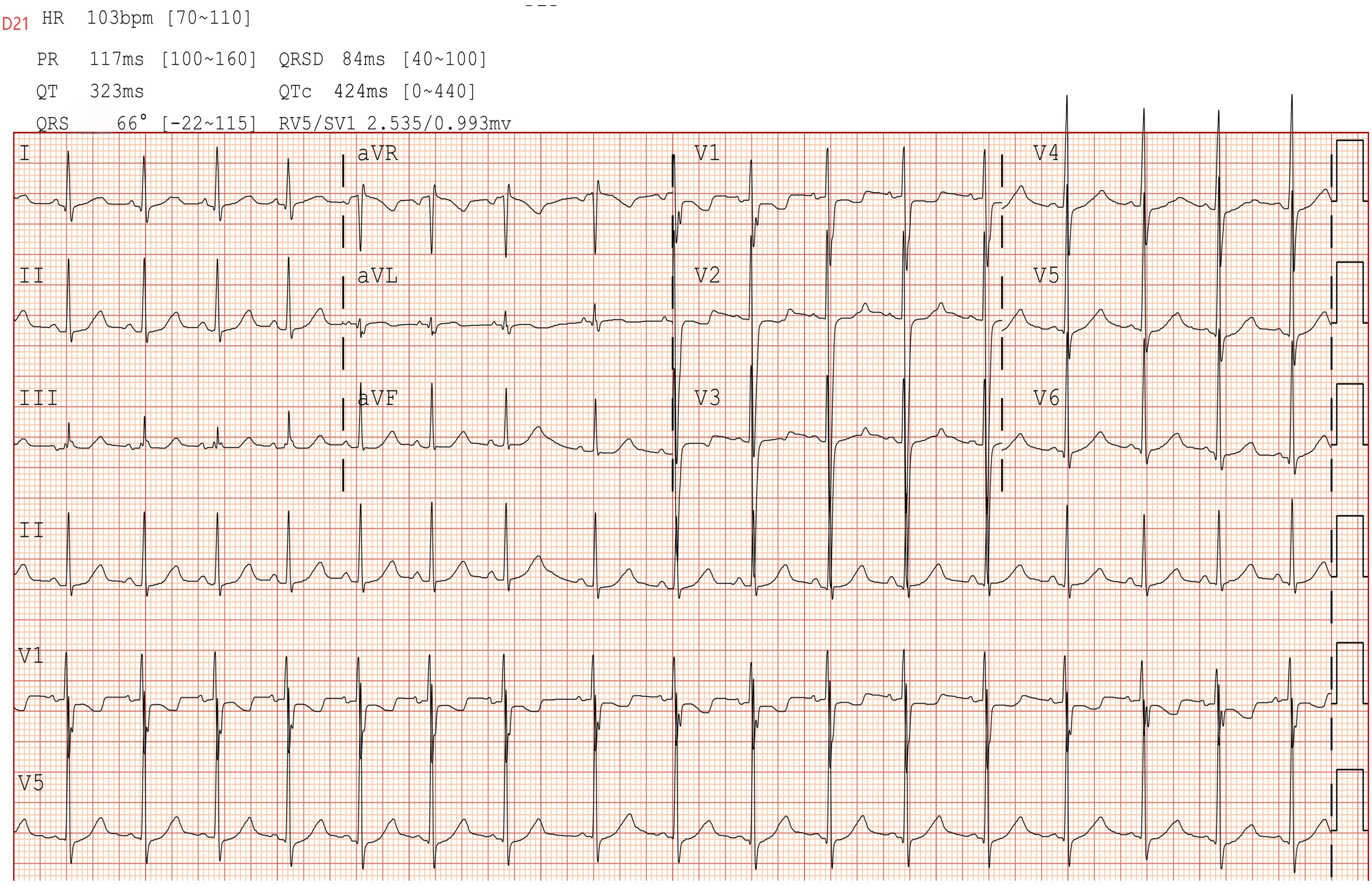

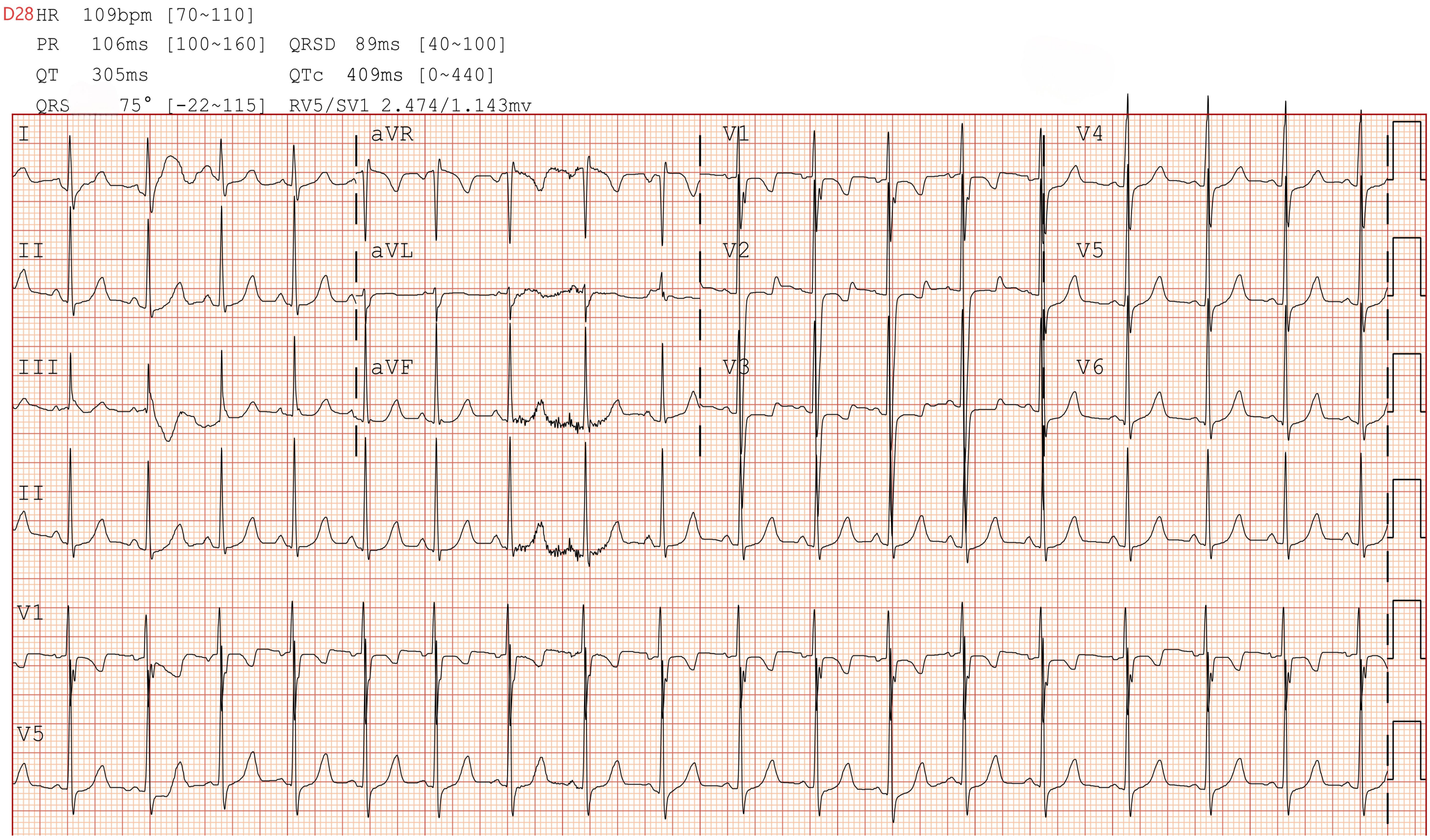

Part 1.Supplementary Material: Serial 12 Lead Electrocardiograms

The figures presents serial standard 12 lead electrocardiograms obtained at key time points during the patient's clinical course. Each tracing is labeled with the corresponding illness day (D2, D16, D17, D20, D21, D28) in the upper left corner, accompanied by detailed ECG parameters

D2: Sinus tachycardia associated with low grade fever during the initial admission.

D16 and D17: Atrial tachycardia is demonstrated. Based on P wave morphology (negative in lead V1, positive in leads I and aVL), the arrhythmia was of left atrial origin, correlating with the concurrent echocardiographic finding of left atrial enlargement.

D20: Restoration of sinus rhythm following termination of atrial tachycardia.

D21: Sustained normal sinus rhythm after deslanoside discontinuation and achievement of complete blood pressure control.

D28: Persistent normal sinus rhythm recorded at the first outpatient follow up visit after the second discharge.

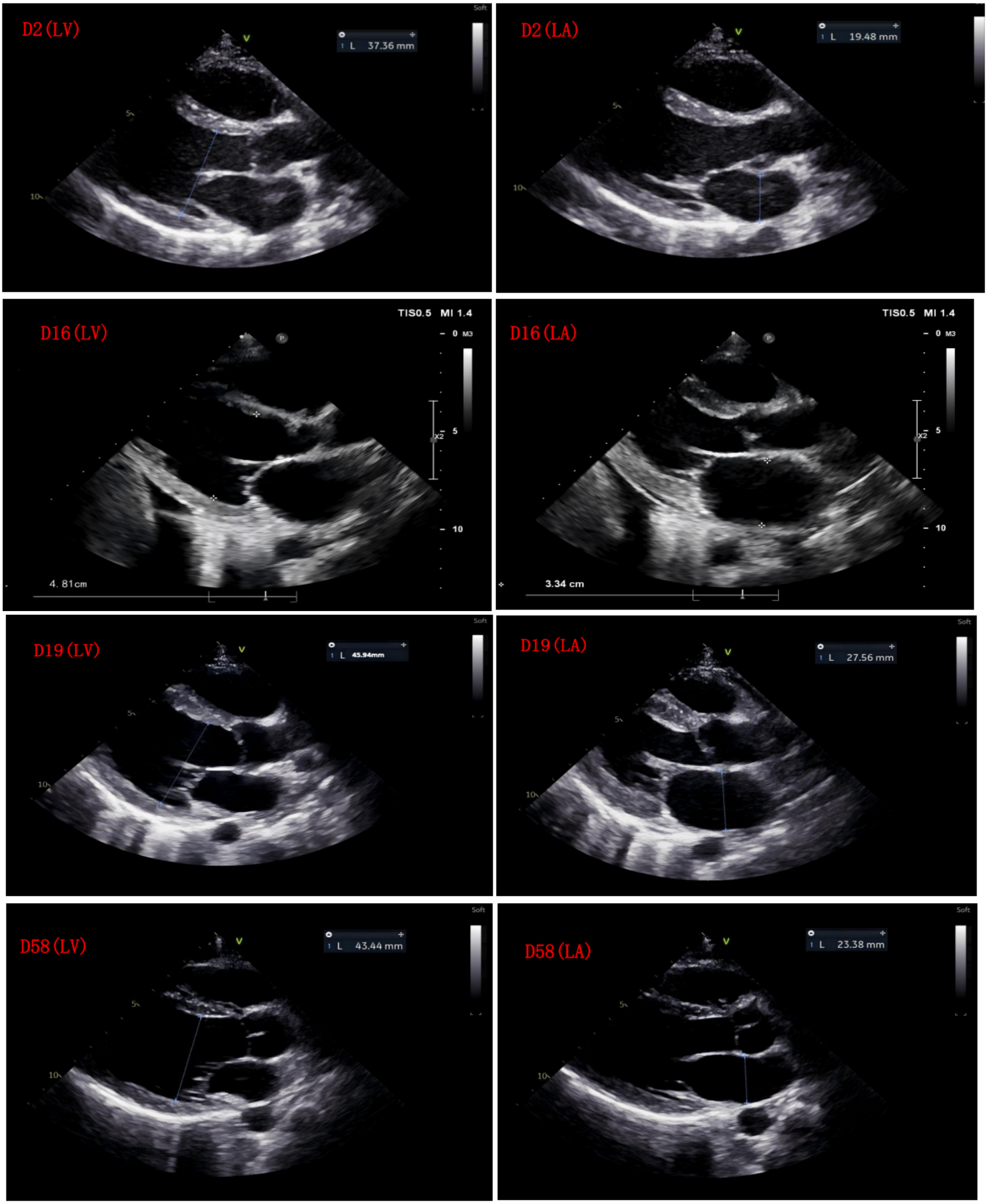

Part 2.Supplementary Material：Echocardiographic Measurements of Left Ventricular and Left Atrial Dimensions.
Standardized measurements obtained from the parasternal long axis view. Left column: Left ventricular end diastolic dimension (LVEDD) measured by two dimensional echocardiography at end diastole, with the measurement line perpendicular to the interventricular septum and left ventricular posterior wall,
extending from the left septal surface to the endocardial border of the posterior wall. Right column: Left atrial anteroposterior dimension (LA) measured at end systole from the trailing edge of the posterior aortic wall to the leading edge of the left atrial posterior wall. The time point (illness day) is indicated in
the upper left corner of each image.
